# Detection of low urine output by measuring urinary biomarkers

**DOI:** 10.1186/s40795-024-00823-3

**Published:** 2024-01-12

**Authors:** Robert G. Hahn

**Affiliations:** Karolinska Institutet at Danderyds Hospital (KIDS), Stockholm, 182 88 Sweden

**Keywords:** Urine analysis, Dehydration, Hydration status, Fluid retention

## Abstract

**Background:**

Urine output < 1 L per 24 h is a clinical warning sign that requires attention from hospital staff, who should determine whether the low flow is due to low habitual intake of water or disease-induced dehydration. The incidence of this condition is unclear.

**Methods:**

A cohort of 20 healthy volunteers (mean age 42 years, range 23–62 years) recorded their food and water intakes daily for 8 days. They also collected and measured all urine and delivered first morning urine samples for analysis of osmolality and creatinine. Optimal cutoffs for these biomarkers to indicate urine output of < 1 L or 15 mL/kg during the preceding 24 h were applied with and without correction for age to cross-sectional data from 1,316 subjects in various clinical settings, including healthy volunteers, preoperative patients, patients seeking acute care at a hospital, and patients receiving institutional geriatric care.

**Results:**

The urine output amounted to < 1 L during 22 of the 159 evaluable study days and was indicated by urine osmolality > 760 mosmol/kg or urine creatinine > 13 mmol/L, which had sensitivity and specificity of approximately 80%. Days with urine output < 1 L were associated with significantly less intake of both water (–41%) and calories (–22%) compared to other days. Application of age-corrected biomarker cutoffs to the 1,316 subjects showed a stronger dependency of low urine output on age than the clinical setting, occurring in 44% of the 72 participants aged 15–30 years and 18% of the 62 patients aged 90–104 years.

**Conclusion:**

Biomarkers measured in morning urine of young and middle-aged volunteers indicated urine output of < 1 L with good precision, but the cutoffs should be validated in older age groups to yield reliable results.

**Trial registrations:**

ISRCTN12215472 at http://www.isrctn.com; NCT01458678 at ClinicalTrials.gov, and ChiCTR-TNRC-14,004,479 at the chictr.org/en.

**Supplementary Information:**

The online version contains supplementary material available at 10.1186/s40795-024-00823-3.

## Introduction

Dehydration impairs physical performance, mood, and cognitive functions [1, 2], but it might be difficult to diagnose through physical examination. Dryness of the mucous membranes, mouth color, and sunken orbits are examples of conditions used in subjective tests for dehydration [3]. Elevated plasma creatinine is a more objective indication, but it requires a previous measurement for comparison. Moreover, an increase is unlikely before dehydration becomes severe. The total intake of fluid, including water in solid food, has been measured in several studies [4–6]. A more practical approach is to measure the urine output, which correlates strongly with water consumption [6–8] and is only occasionally due to acute dehydration [9]. Very low urine output can also serve as an early sign of ongoing disease [10, 11].

The frequency of low urine output in different populations is largely unknown. Urinary biomarkers such as osmolality and creatinine were first used to detect dehydration in elite athletes [12]. Short-term changes have been related to concomitant decreases in body weight, but cross-sectional studies have usually involved only one urinary sample and no known change in body weight [13]. Using this information, the relationships between biomarker concentration, water consumption, and urine output can be calculated through linear regression [7].

Urine output of 1 L is a practical clinical cutoff as lower output warrants attention from a medical team and clarification of the reasons for the low urine flow. Possible explanations include poor fluid intake, outflow obstruction, or acute kidney injury. Subjects defined as “low drinkers” excreted 1.01 L of urine per 24 h in a study by Perrier et al. [8]. A rise in the plasma concentration of vasopressin to 2 pg/mL is the brain’s signal to initiate water conservation and occurs when the total water intake per 24 h falls below 1.8 L [14], which is expected to produce a urine output of 1.1–1.2 L (60–70% of the water intake) [6].

A study on urinary biomarkers at 30-min intervals during surgery shows that the urine-specific gravity begins to increase when its flow is < 1 mL/min, which corresponds to 1.4 L/24 h [15]. Therefore, urine output of < 1 L in an adult human implies an increased plasma concentration of vasopressin and that the kidneys are conserving water. Apart from an acute healthcare setting, the detection of low urine output is relevant in population studies as mild dehydration is associated with several acute and chronic diseases that are positively affected by improved hydration status [14].

In young people, biomarkers measured the first morning urine can predict the hydration practices during the preceding 5 days and offer a cost-effective tool for the detection of habitual underhydration [16]. The aim of the present study was to find out more precisely how well a urine sample taken in the early morning can provide information about low urine output on the previous day. For this purpose, data from a diet study were used to identify optimal cutoff values for urine osmolality and creatinine measured in the first morning urine to indicate when < 1 L of urine was excreted during the preceding 24 h [6]. This volume corresponds to approximately 15 mL/kg in humans who are not severely under- or overweight. The optimal cutoffs were used to study the incidence of daily urine outputs of < 1 L in 1,316 volunteers and clinical patients with a wide range of ages [17–24].

The hypothesis was that older persons admitted for elective surgery or geriatric care have a higher incidence of low urine output than healthy young people.

## Methods

### Ethics approvals

The protocol for the **diet study** had been approved by the Regional Ethics Committee of Stockholm (June 15, 2016, Dnr. 2016/826 − 31, Chairperson Hans Glaumann) and registered in an international database, http://www.isrctn.com, as identifier ISRCTN 12,215,472.

The protocols for the **cross-sectional studies** were approved by the Regional Ethics Committee of Stockholm, Sweden (2009/1838-31/2, 2009/1675-32, 2010/1128-31, 2013/380 − 31/3, 2013/903 − 31/1, 2014/497 − 31/4**).** the Regional Ethics Review Board of Linköping, Sweden (2011/101 − 31), and the Ethics Committee of the First Affiliated Hospital, College of Medicine, Zhejiang University, China (Dnr. 2,011,150). Database registrations were made at the ClinicalTrials.gov (NCT01458678) and the Chinese Clinical Trial Registry (chictr.org/en; ChiCTR-TNRC-14,004,479).

Detailed descriptions of the patient groups and sampling conditions are given in the respective studies. Due to occasional missing data, there may be minor differences between the number of patients mentioned in the original studies and those reported here. The presentation follows the STROBE checklist.

### Diet study

The optimal biomarker cutoffs indicating urine volume < 1 L over 24 h were derived by retrospective analysis of a diet study. The prospective non-randomized diet study involved 20 hospital workers who volunteered to participate during two weeks between November 2016 and March 2017 [6]. The volunteers ingested fluids as usual during the first week of the study. On the first four days, all urine excreted during each 24 h (from 7 AM to 7 AM) was collected, measured, and sampled. Every morning, before any fluid or food was ingested, each volunteer also took a sample from the first portion of urine voided and delivered it for analysis to the hospital’s clinical chemistry laboratory.

During the second week, the volunteers were instructed to ingest one additional glass of water with each meal, which resulted in an increase of total water consumption by 32%. During this time as well, excreted urine was collected and measured for each 24 h during the first 4 days. The volunteers provided spot urine samples at any time of the day (whenever they experienced urgency) on the other days. These spot samples were analyzed for biomarkers and the results reported elsewhere [25], but the urine volumes were not measured.

The volunteers weighed all ingested liquids and foodstuffs on a scale and recorded the type of food either via a physical list or an online program. Duplicate weighting was not employed. The water and nutritional content for each consumed foodstuff was calculated for each 24 h by a dietitian using the software Dietist Net (http://www.kostdata.se; Kost och Näringsdata, Bromma, Sweden). The dietician also personally instructed each participant about how to weigh and record the food before the study started. The reported water intakes included both liquid and the water content of the food. At the end of the first week, the body weight was measured on an electronic scale, and the extracellular and intracellular body fluid volumes were estimated by multifrequency bioelectrical impedance (Xitron 4000B, Xitron Technologies Inc., San Diego, CA, US) [26].

### Cross-sectional studies

Data on urinary biomarkers of renal water conservation were obtained from 1,316 males and females, who were divided into four groups according to their clinical situation and age range. Studies were chosen to obtain a wide coverage of different scenarios that could provide information about urine biomarkers and associated low urine output (< 1 L) in sensitive sub-populations, such as care-seekers and hospital patients. The author planned and supervised all the included studies.

Participants in Groups 1 and 2 were asked to abstain from intake of liquid from 2 h before the urine sampling. The other groups had no water or food restrictions.

#### Group 1

Comprised *volunteers*. In this group, 55 urine samples were obtained in the early evening from healthy people before performing recreational jogging (median time 90 min of tennis, jogging, or Thai boxing) after their working day [17]. Moreover, 300 urine samples were obtained at daytime from hospital staff [18].

#### Group 2

Comprised *patients just before surgery*, among which morning urine samples were collected from 110 patients on the morning before elective abdominal surgery [19], from 41 patients scheduled for acute hip fracture surgery [20], and 106 patients who were to undergo colorectal cancer surgery [21]. A quality assurance program based on the ethics permit for Group 3 consisted of 24 samples collected on the morning before elective orthopedic surgery.

#### Group 3

Comprised *patients seeking acute care.* 311 patients delivered a urine sample in the daytime while awaiting evaluation at the Acute Care Department of Södertälje Hospital.

#### Group 4

Comprised individuals receiving *geriatric care in a hospital.* Morning urine was sampled from 256 patients who had just been admitted to the hospital for acute geriatric care [22], as well as 55 patients from a nursery home [23], 23 elderly patients with heart failure, and 25 without heart failure who had been admitted for hospital care but of the same age as those with heart failure (mean, 80 years) [24].

The exclusion criteria in the diet study were professional sports activities and any disease that required daily medication or a special diet. The exclusion criteria in the cross-sectional studies were inability to void, seriously impaired cardiac function, and impaired kidney function requiring hemodialysis.

### Urine measurements

The urine osmolality and the urinary creatinine concentration were measured within 36 h after sampling with an Advanced 2020 osmometer (Molek AB, Sweden) and Cobas 8000 analyzer (Roche Diagnostics, Basel, Switzerland), respectively. The measurements were done at the certified clinical laboratory at Karolinska University Hospital in Stockholm. The coefficient of variation was 3% for osmolality and 5% for creatinine (at 6 mmol/L). The volunteers were not aware of the results of any analysis during the experimental period.

### Corrections for age

The renal capacity to concentrate urine is expected to decrease by 5 mosmol/kg per year beyond the age of 20 years [27, 28]. The lower limit of urine osmolality for hypohydration decreases according to the following: ≥ 831 mosmol/kg– (3.4 (age– 20 years) [29]. A population study of US citizens who were not water deprived showed that the urine osmolality decreases by 2.9 mosmol/kg per year [30].

### Exploratory analyses

The results of the cross-sectional studies motivated inclusion of additional variables that could strengthen the conclusions, although these variables had not been consistently measured. Plasma creatinine was measured in the hospital’s routine laboratory in 504 patients from Groups 2, 3, and 4. The urine albumin/creatinine ratio was measured bedside at the time of the urine sampling in 480 patients using a DCA Vantage Analyzer (Siemens Healthcare Diagnostic, Erlangen, Germany). The degree of perceived thirst had been quantified in 208 patients using a visual analogue scale where 0 implied no thirst and 100 implied maximum thirst.

### Statistics

Results are reported as the mean ± standard deviation (SD), and differences between subgroups were evaluated by one-way analysis of variance followed by the Scheffé *post hoc* test. Differences in nominal distributions were studied using the chi-square test. Data showing a skewed distribution are reported as the median (25th–75th percentile limits), and differences between the study groups were assessed using the Kruskal-Wallis test followed by the Jonckhere-Terpstra *post hoc* test to determine between-group differences. Multiple regression analysis was used to examine linear relationships between continuous variables, and logistic regression analysis was used to identify the variables that most strongly predicted lower urine output (< 1 L / 24 h and < 15 mL/kg per 24 h).

Receiver operating characteristic (ROC) curves were used to express the ability of ranges of biomarkers to determine whether the excreted urine volume during the preceding 24 h had amounted to 1 L. ROC curves are probability curves where sensitivity (true positive fraction) is plotted versus 1– specificity (false positive fraction). The area under the curve (AUC) for this relationship reflects how well ranges of biomarkers can be separated. The prediction of the probability of the binary outcome given by the ROC curve (i.e., the urine volume being greater or smaller than 1.0 L) is statistically significant if the 95% confidence interval does not include 0.5. SPSS version 25 (IBM) for MacOS was used for the data analysis and calculations.

## Results

### Diet study

Demographic data and the main results of the diet study are shown in Table 1. The evaluation was based on 159 morning urine samples that were matched with 24-h collections of urine (data from one day were incomplete). The volume of 22 urine collections from 9 volunteers was < 1 L. The urine volume was > 1 L in 137 collections. Only 5 urine volumes amounted to < 1 L during the second week, when the mean fluid intake was 32% higher than during the first week.

**Fig. 1:**
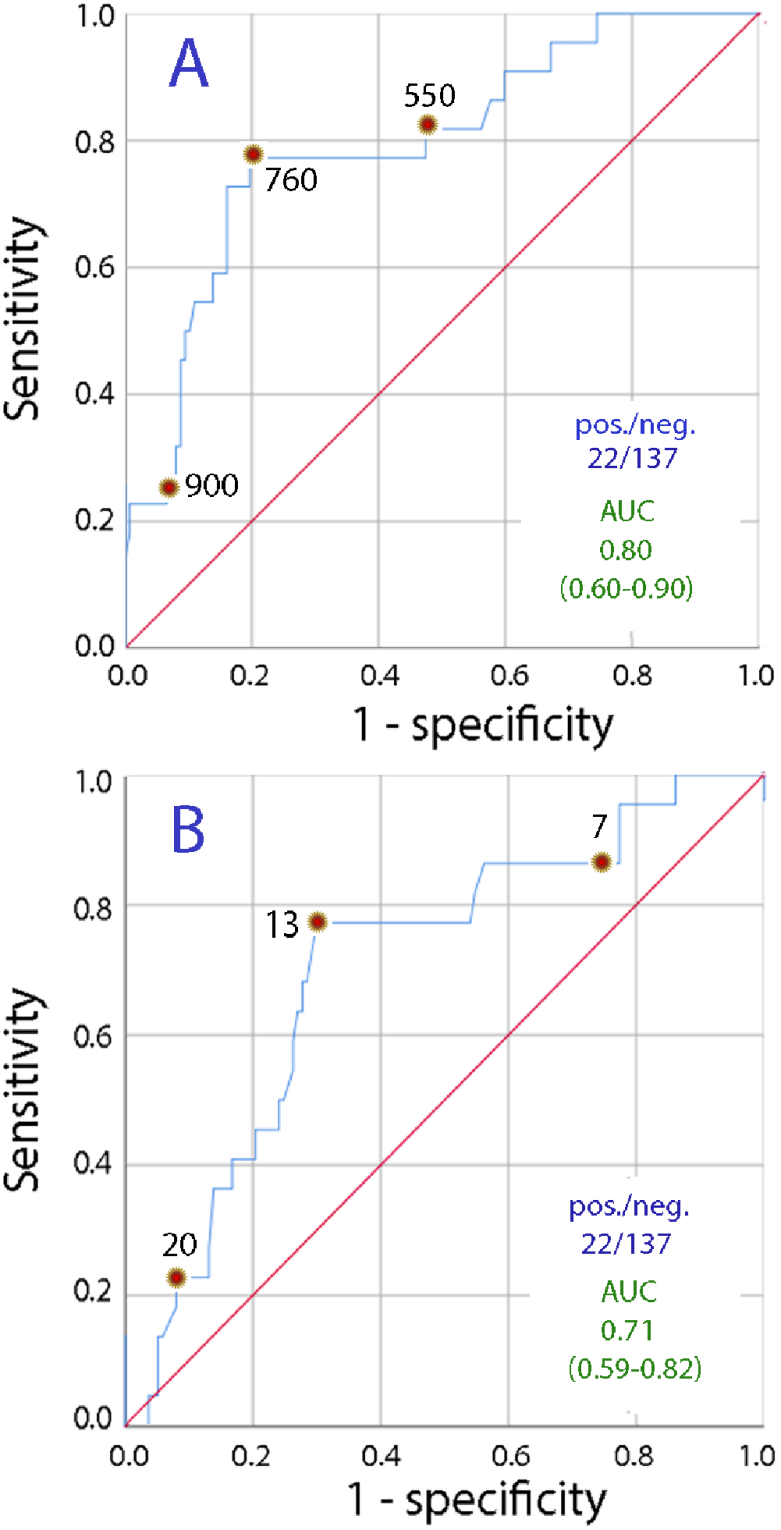
Fixed urine output. Receiver operating characteristic (ROC) curves showing cutoff points where (**A**) urine osmolality and (**B**) urine creatinine concentration measured in the first voided morning urine indicate that the excreted urine during the preceding 24 h had a volume of < 1 L. Selected cutoff points are marked

The osmolality in the morning urine was 805 ± 191 mosmol/L when the urine output during the preceding day was < 1 L, while the osmolality in the other morning samples amounted to 571 ± 190 mosmol/kg (*P* < 0.001). The corresponding data for urine creatinine were 14.6 ± 5.2 and 10.8 ± 5.6 mmol/L, respectively (*P* < 0.005). The ROC curves suggested that a urine osmolality of > 760 mosmol/kg indicated a urine volume of < 1 L during the preceding 24 h with sensitivity and specificity of approximately 80% (Fig. 1A). The urinary creatinine concentration was a slightly poorer biomarker, but concentration > 13 mmol/L indicated urine output < 1 L with a precision of almost 80% and specificity of 70% (Fig. 1B).

**Fig. 2:**
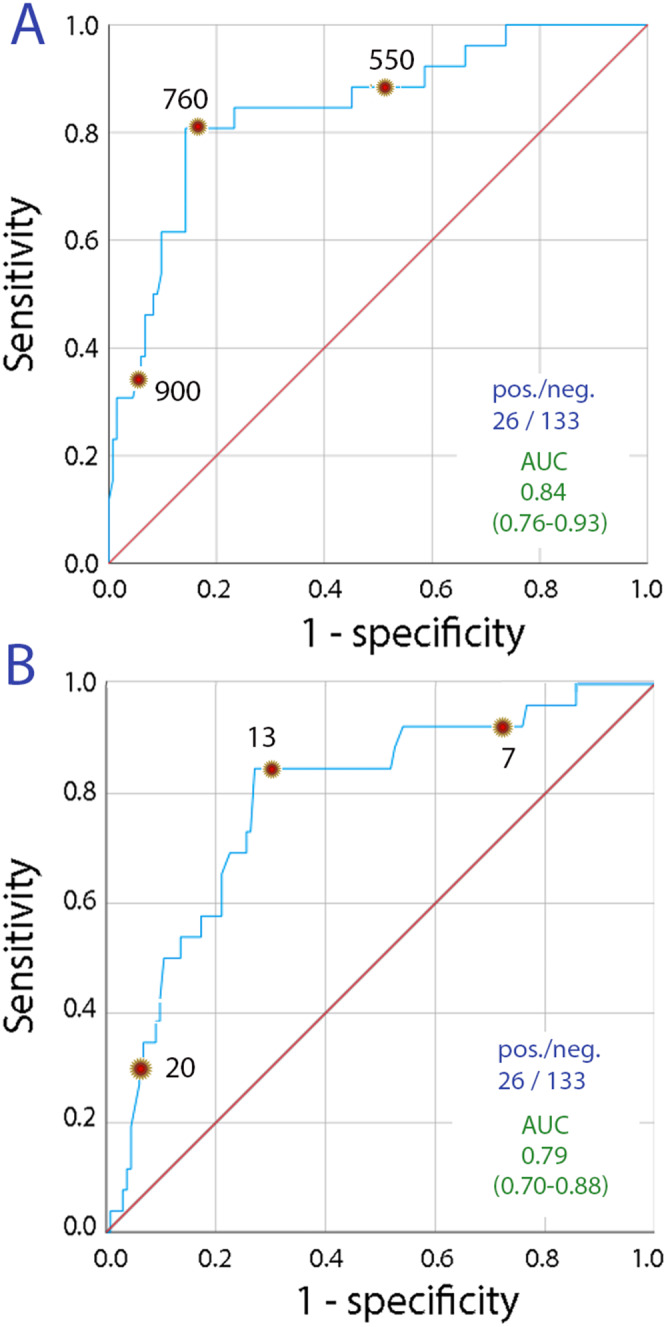
Urine output corrected for body weight. The same ROC plots as in Fig. 1 but using a cutoff of < 15 mL/kg for the urine output during the preceding 24 h

Analysis of the goodness of fit indicated that the optimal cutoff for osmolality (chi-squared, 31.3) was more effective than the cutoff for creatinine (chi-squared, 16.9) at indicating urine volumes < 1 L. Combining the two biomarkers did not appreciably improve the precision by which urine volumes < 1 L could be indicated. The bioimpedance measurements did not reveal any statistically significant difference in extracellular or intracellular body fluid volume between those who voided < 1 L and > 1 L (Table 1). ROC curves were also constructed for using biomarkers measured in the morning urine to detect urine volumes < 15 mL/kg during the preceding day (Fig. 2). The urine output corrected for body weight offered marginally better precision than the crude urine volume, but the optimal cutoffs were virtually identical (urine osmolality > 760 mosmol/l and urinary creatinine > 13 mmol/L).

With correction for body weight, the osmolality in the morning urine was 825 ± 172 mosmol/L when the urine output was < 15 mL/kg L while it was 560 ± 84 mosmol/kg for other cases (*P* < 0.001). The corresponding data for urine creatinine were 16.2 ± 5.4 and 10.4 ± 5.3 mmol/L, respectively (*P* < 0.001). Logistic regression analysis was used to identify the variables that most strongly predicted low urine output. Statistically significant predictors in the univariate analysis are shown in Table 2. Multivariate analysis indicated that water intake (*P* < 0.001) and the morning urine osmolality (*P* < 0.04) were the only statistically significant predictors of urine output < 1 L/24 h as well as < 15 mL/kg/24 h.

**Table 1:** Demographic data and body fluid volumes for volunteers who voided < 1 L on any of the 8 days that urine was collected. The diet data represent the values obtained on each day that the urine volume was < 1 L. Data are the mean (SD) except where noted

	Urine < 1 L	Urine > 1 L	Significance
Demographic data			
Age (years)	41 ± 12	43 ± 11	0.68
Body weight (kg)	69 ± 12	75 ± 9	0.21
Female/male (N)	8 / 1	8 / 3	0.37 ^1^
Diet data			
Water intake ^2^ (L)	1.81 ± 0.47	3.07 ± 1.11	0.001
Liquid intake (L)	1.30 ± 0.45	2.38 ± 1.07	0.001
Energy, total (kcal)	1,587 ± 502	2,047 ± 637	0.002
Protein (g)	68 ± 22	87 ± 29	0.005
Fat (g)	69 ± 32	94 ± 38	0.004
Carbohydrates (g)	159 ± 89	188 ± 95	0.18
Fibers (g)	11 ± 7	15 ± 7	0.013
Body fluid volumes			
ECV (L/kg body weight)	0.221 ± 0.020	0.216 ± 0.014	0.77
ICV (L /kg body weight)	0.291 ± 0.050	0.278 ± 0.046	0.51
Biomarkers, urine			
Osmolality morning (mosmol/kg)	805 ± 191	571 ± 190	0.001
Creatinine, morning (mmol/L)	14.6 ± 5.2	10.8 ± 5.6	0.001

ECF = extracellular fluid space; ICV = intracellular fluid space

^1^ chi-square test was applied, ^2^ sum of liquid and food water

**Table 2:** Univariate logistic regression analysis results identifying the variables that most strongly predicted low urine output. Each day is one measurement (*N* = 159). A positive sign means that a high value of the variable is associated with a higher incidence of low urine output

	< 1 L / 24 h				< 15 mL / kg / 24 h		
	Score	P-value	+ / -		Score	Significance	+ / -
Water	23.4	0.001	-	U-osmolality	32.3	0.001	+
Liquid	21.7	0.001	-	Water	32.2	0.001	-
U-osmolality	20.4	0.001	+	Liquid	29.2	0.001	-
Protein intake	9.2	0.002	-	U-creatinine	19.8	0.001	+
Calories	8.9	0.003	-	Cardiac index ^1^	17.1	0.001	+
Fat intake	7.9	0.005	-	Protein intake	9.2	0.002	-
Age	7.2	0.007	+	Intake of fibers	5.0	0.034	-
U-creatinine	6.6	0.010	+	Calories	4.7	0.031	-
Body weight	4.6	0.031	-	Fat intake	4.5	0.034	-

^1^ cardiac output/body surface area; measured with Nexfin non-invasive hemodynamic monitor (BMEYE, Amsterdam, NL)

### Cross-sectional studies

**Fig. 3:**
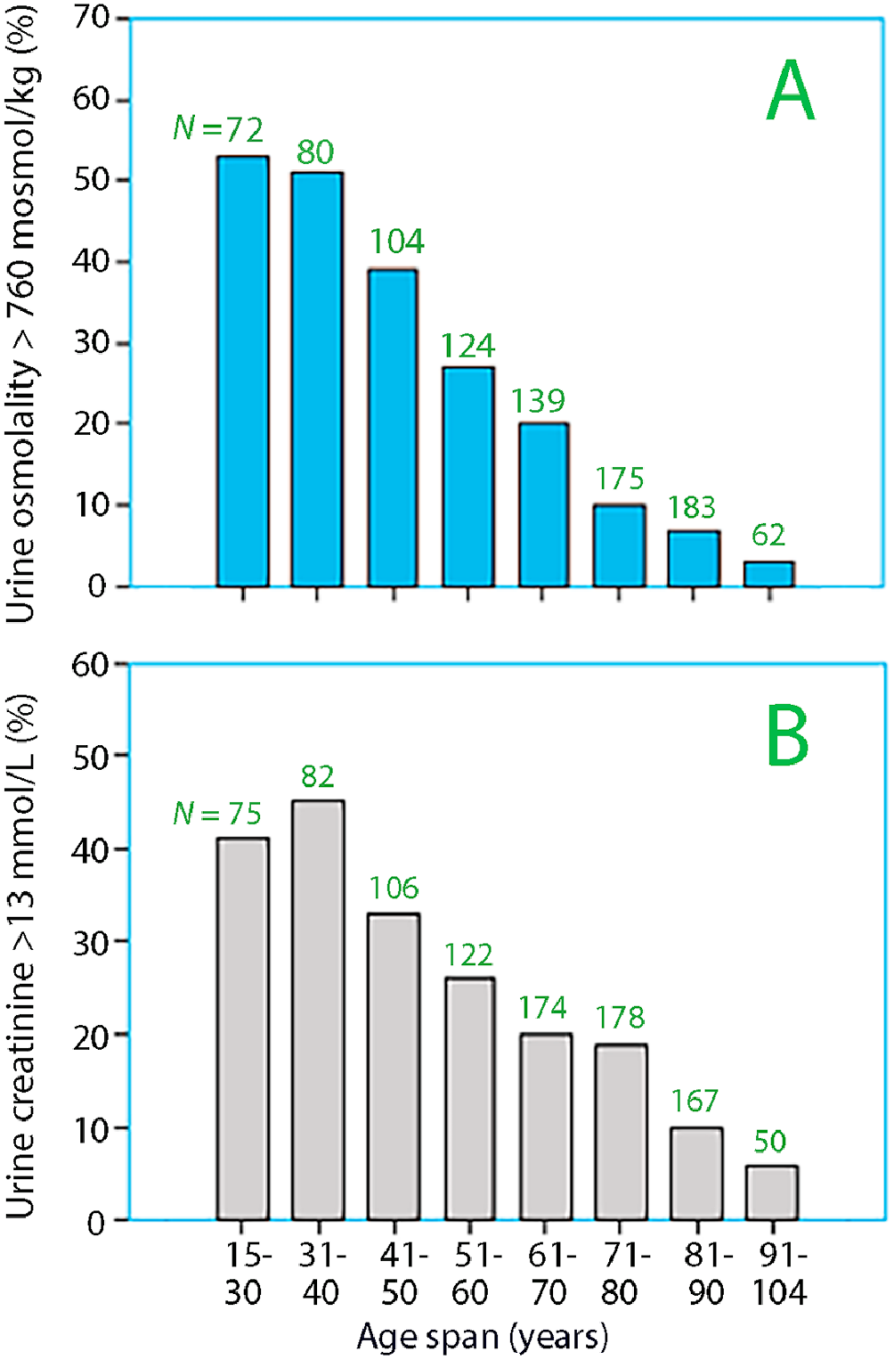
The incidence of low urine output (< 1 L per 24 h) for different age groups in 10 cross-sectional studies based on (**A**) urine osmolality and (**B**) urine creatinine concentration measured in the morning urine. The number of participants is shown on top of each bar

Demographic data and the results of the urine analyses in the cross-sectional studies are shown in Table 3. The proportion of volunteers in Group 1 who voided < 1 L was 42% when applying a cutoff of 760 mosmol/kg for urine osmolality. The patients scheduled for surgery (Group 2) had an incidence of 20%, while the patients who were admitted to acute geriatric care in hospital (Group 4) had an incidence of only 5%. Urine creatinine indicated low urine output in 33% of the volunteers (Group 1), 21% of the preoperative patients (Group 2), 17% of those seeking acute care (Group 3), and 8% of the geriatric patients (Group 4). The incidence of concentrated urine suggesting urine output < 1 L decreased with age when indicated by both urine osmolality (Fig. 3A) and urine creatinine (Fig. 3B).

### Corrections for age

The maximum urine osmolality would average 832 mosmol/kg in the highest age group (90–104 years) if the kidneys were able to concentrate the urine to 1,200 mosmol/kg at the age of 20 years, and the capacity would decrease thereafter by 5 mosmol/kg per year [27, 28]. The measured urine osmolality in the oldest patients was 370 (238–429) mosmol/kg below this maximum value, and those aged between 80 and 90 years had urine osmolality that was 407 (305–500) mosmol/kg below their maximum capacity. Application of the equation for hypohydration by Manz and Wentz [29] to the data indicated that the incidence of low urine output was 42% in the volunteers (Group 1), 26% for patients before surgery (Group 2), and 20% in geriatric patients (Group 4).

**Fig. 4:**
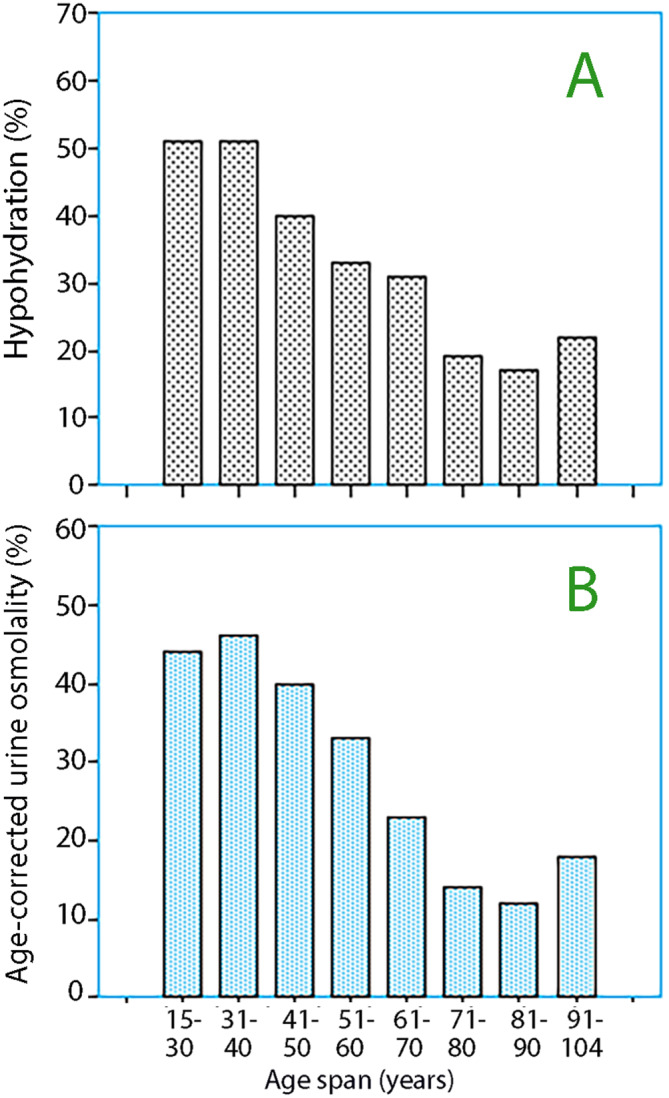
(**A**) Incidence of hypohydration for different age groups when urine osmolality is corrected according to Manz and Wentz [29]. (**B**) Incidence of low urine output when urine osmolality is corrected by 2.9 mosmol/kg per year in participants aged > 40 years. The cutoff for low urine output was 760 mosmol/kg after correction. The number of participants is the same as in Fig. 2A

The incidence of hypohydration decreased with age (Fig. 4A). Age correction of the urine osmolality by 2.9 mosmol/kg per year [30] starting at the mean age of the diet study (40 years) indicated an incidence of low urine output of 48% in the volunteers, 26% in the preoperative patients, and 17% in the geriatric patients. Hence, the incidence of low urine output decreased with age despite correction for age (Fig. 4B).

**Table 3:** Urine biomarkers of renal water conservation in the four groups of subjects. Data are the median (25th -75th percentile range)

	Volunteers	Just before surgery	Seeking acute care at hospital	Geriatric care	ANOVA	Scheffé Test *P* < 0.05
Group Nr	1	2	3	4		
N	354	286	316	360		
Age (years)	43 (32–54)	66 (56–74)	80 (73–86)	83 (77–89)	0.0001	All differ
Body weight (kg)	71 (62–80)	67 (58–79)	-	69 (57–79)	0.034	1 < 3, 4
Urine osmolality (mosmol/kg)	686 (486–893)	543 (378–705)	-	454 (364–589)	0.0001	All differ
Urine creatinine (mmol/L)	10.1 (6.4–14.6)	7.5 (4.8–12.1)	6.6 (3.9–10.9)	7.6 (5.6–9.7)	0.0001	1 < 2, 3
Urine < 1 L (%)						
U-osmolality	42%	20%	-	5%	0.0001	1 > 2 > 3
U-creatinine	33%	21%	17%	8% ^1^	0.0001	1 > 2–4
Agreement	81%	82%	-	85%	-	-
Males / females						
U-osmolality	54% / 36%	27% / 13%	-	10% / 2%	-	
U-creatinine	45% / 28%	25% / 17	21% / 14%	6% / 9%	-	
U-albumin / U-creatinine ratio (mg/mmol/L)	0.7 (0.5–1.1)	2.1 (0.9–7.1)	3.8 (1.6–11.5)	2.4 (1.2–4.7) ^1^	0.0.0001 ^2^	1 < 2–4, 3 > 2, 4
Thirst (VAS scale 0-100)	21 (14–38)	40 (20–50)	-	30 (21–45).	0.0.0003 ^2^	1 < 2, 4

^1^ only 52 measurements were available. ^2^ Kruskal-Wallis test was applied followed by the Jonckhere-Terpstra *post hoc* test

### Exploratory analyses

**Fig. 5:**
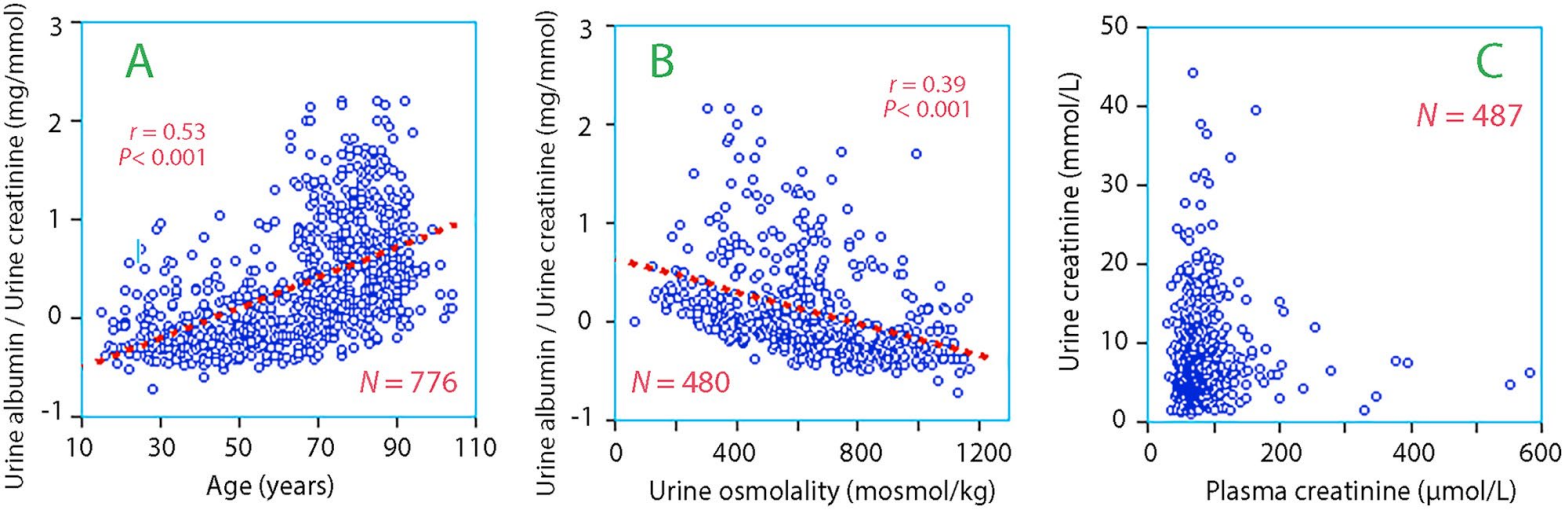
(**A**) Increase in albuminuria with age. (**B**) Decrease in albuminuria with higher urine osmolality. Micro-albuminemia (2.5 mg/mmol) is present when y > 0.4 on the log-scale. (**C**) Plasma versus urine creatinine. Massive overlapping

Albuminuria increased with age (Fig. 5A) and was associated with lower urine osmolality (Fig. 5B) and urine creatinine (not shown). Micro-albuminuria (defined as > 2.5 mg/mmol creatinine) was present in < 10% of the subjects younger than 50 years, while those older than 60 years had an incidence of > 50%. High plasma and urine creatinine appeared to be mutually exclusive (Fig. 5C).

Perceived thirst did not change with age, and only 25% of the observations in each age group had a rating that exceeded 50 on the scale ranging from 0 to 100. The median values per age group (as defined in Figs. 3 and 4) were 36 (*N* = 16), 31 (*N* = 16), 30 (*N* = 19), 26 (*N* = 38), 30 (*N* = 39), 40 (*N* = 41), 35 (*N* = 28), and 30 (*N* = 9). Multiple regression analysis did not reveal any significant linear relationship between the thirst ratings and urine biomarkers (*N* = 188). The thirst ratings were even lower when urine osmolality indicated low urine output (27 ± 19 vs. 37 ± 22; *P* < 0.012).

High intake of protein is known to increase solute excretion [14]. The total excretion of solutes and creatinine per 24 h in the diet study are available in Supplementary File 1.xls and have been reported in detail in a previous study [6]. In the diet study, volunteers with urine output < 1 L/24 h had a lower excretion of solutes (-30%) and creatinine (-23%) compared to those who voided > 1 L per 24 h (both, *P* < 0.001). Multiple linear regression confirmed that the solute excretion per 24 h increased with the protein consumption (*P* < 0.001) but not with the intake of carbohydrates or fat. The excretion of creatinine per 24 h correlated positively with the ingested amount of protein and carbohydrates (both *P* < 0.001) and inversely with the intake of fat (*P* < 0.04).

## Discussion

### Key result

The results of the diet study confirm that excreted urine volumes < 1 L over 24 h can be estimated with good confidence in middle-aged volunteers by analyzing biomarker levels in urine sampled in the early morning one day later. The cutoff values for the biomarkers were quite similar when the excreted urine volume was corrected for body weight, but the strength of the correlations was marginally stronger. Low urine output was associated with low intakes of water, calories, protein, fat, and fibers. In contrast, the body weight and the body fluid volumes were the same as in volunteers who excreted more urine than 1 L/day, which shows that the kidneys were effective in conserving water when the water intake was lower.

The optimal cutoffs for the biomarkers derived in the diet study were applied to data from 1,316 subjects derived from 10 cross-sectional studies performed or supervised by the author. The psychological and disease-induced stress associated with hospital settings was believed to promote stronger fluid retention compared to volunteers. However, the incidence of low urine output was more dependent on age than on clinical situation. The incidence decreased sharply with age in a way that could not be fully explained by the known age-dependent reduction of the renal concentrating capacity [31–34]. The interpretation was complicated by the stunningly high frequency of low urine output among the volunteers, who were mostly hospital workers. Therefore, the study hypothesis was refuted based on the method used.

Additional findings include a high frequency of albuminemia after 60 years of age, which suggests that the osmolar excretion in the elderly could have been troubled by vascular damage to the glomeruli. Albuminuria increases the renal clearance of infused electrolyte-rich fluid [35], which is counteracted in younger people by a more intensive concentration of urine [18]. However, aged kidneys may not be able to meet this challenge, resulting in dehydration despite normal or low urine osmolality.

### Fluid balance versus urine analysis

Recording the water consumption with high accuracy is difficult. Intakes of liquid can be overlooked, and healthcare workers often estimate the ingested volumes differently [36]. New technical solutions are available [37] but the contribution of water from food is still difficult to estimate.

Measuring urine output might be a shortcut to understanding whether a patient has a problem with the fluid balance. Urinary excretion accounts for 60–70% of the total intake of water and to 85% of the ingestion of liquid [6]. However, collecting all urine over 24 h is prone to errors. A simple urine test that identifies inadequate intake of water is less labor-intensive and could be of great value to hospital staff and scientists performing population studies of body hydration. Urine osmolality has even been thought to be a more objective measure of hydration status than self-reported fluid intake [38]. Urine analysis has established value when diagnosing electrolyte and acid-base disorders [39], but its use for screening health-status issues in public and clinical medicine is limited due to uncertainty about the validity, interpretation, and precision.

Studies performed during the past decade highlight the great variation in urine biomarker levels in the population [18] and point out the strong relationship between these variations and the habitual ingestion of water [6–8, 40]. Therefore, the concentrated urine reported in the diet study is unlikely to indicate blunt dehydration but rather illustrates the effectiveness of renal adaptation to variations in fluid intake. On the other hand, the results of the cross-sectional studies suggest that measurements of biomarkers in the urine cannot be uncritically accepted to indicate the fluid balance in older age groups.

A concern when analyzing morning urine is that the concentrations of biomarkers require several days to show marked changes when the habitual intake of fluid is increased, particularly when increased from a low level. Doubling the fluid intake reduces urine osmolality [40–42], but the smaller increase in fluid intake in the present diet study (+ 32%) only slightly diluted the biomarkers [6]. Therefore, the second week contributed only 23% of the correct indications and 61% of the false indications of urine output < 1 L when using the osmolality cutoff of 760 mosmol/kg. The argument for still including the second week is that the reported sensitivity and specificity of the urine analyses should include a certain moment of abrupt change in water intake, as it might occur in daily life.

### Dehydration in the elderly

The present study focuses on low urine output as a sign that requires further diagnostic attention. Low urine output is easier to define than dehydration. A Cochrane review from 2015 examined 71 different indices of dehydration in people older than 65 years. Only three of them were found to be clinically useful: missing drinks between meals, expression of fatigue, and bioimpedance resistance at 50 kHz [43]. Fluid intake and urine output were not found to be useful even though mismatch between these variables is the foundation of dehydration in the absence of extreme heat and diarrhea.

A problem in studies on dehydration is the identification of the correct answer. The Cochrane analysis used plasma/serum osmolality or a rapid decrease of body weight as the only valid evidence of dehydration. This definition is probably too narrow. Impaired renal function might interfere with the available diagnostic tools. Data from 98 nursery-home patients (mean age 83 years) from our institution [23, 44] were reviewed along with 40 patients from an internal report. The results identified a ^10^log increase of the plasma creatinine concentration as the key variable correlating with hyperosmolality (≥ 300 mosmol/kg in serum). The 64 patients with hyperosmolality could have urine osmolality anywhere between 250 and 950 mosmol/kg. Fluid intake averaged 1.2 L regardless of whether hyperosmolality was present, which is half of the recommended intake of water [4, 5]. These observations point out the need for longitudinal studies of water intake and urine output in the elderly.

Another issue is that hyperosmolality is a late sign of dehydration. Popowski et al. found that serum osmolality ≥ 300 mosmol/kg corresponded to a loss of 5% of body weight [12]. Cheuvront et al. reported that serum osmolality reached 297 mosmol/kg when 2% of the body weight had been lost [45]. In contrast, a change in urine osmolality might detect loss of 1% of the body weight [17]. These evaluations have been performed with healthy volunteers subjected to exercise-induced acute dehydration, but applicability in older in-hospital patients is unclear.

### Strengths and limitations

The present study of the incidence of low urine output in different populations included volunteers, patients awaiting surgery and acute care, and in-hospital geriatric patients. The ability of the urinary biomarkers to detect low urine output in the middle-aged volunteers in the diet study was good, reaching sensitivity and specificity close to 80%, but questions must be raised regarding their applicability in the other settings. The strengths of the diet study include its use of volunteers who were hospital workers (usually nurses), who have experience of following a protocol. All data collection was performed in a standardized way. All consumed fluid and food were weighed on an electronic scale. The water present in ingested foodstuffs was calculated by a dietician and included in the analysis. All biochemical measurements were performed within 36 h, even on weekends, by an accredited hospital laboratory.

The limitations include that the number of participants in the diet study was small, although each of them contributed with 8 matches between urine volume and biomarker measurements. Another limitation is that 16 of the 20 participants were women. The variance of the water consumption was deliberately increased by inviting volunteers to the study who presented with either very dilute or concentrated urine in an initial screening [25].

The definition of urine output of < 1 L as “low” is arbitrary. Reference can be given to the coinciding of the excretion of < 1 mL/min (i.e., < 1.4 L per 24 h) with rising specific gravity of urine in women undergoing open hysterectomy [15]. Hence, urine volumes < 1 L per 24 h imply that the kidneys concentrate the urine to maintain the body fluid volumes. Moreover, the cutoff value of 760 mosmol/kg that we derived in the diet study, where the mean age was 42 years, is almost identical to the cutoff for hypohydration defined by Manz and Wentz [29]. The cutoff at 1 L also divided the participants into two groups that had similar water ingestion, urine output, and urine osmolality to the study by Johnson et al. [40]..

Muñoz and Bergeron [16] recently reported that biomarkers measured in the first morning urine show high consistency over time and adequately reflect the biomarker concentrations, total water intake, and the plasma concentration of co-peptin (precursor of vasopressin) measured during the preceding 5 days. In the present study, the first morning urine was usually sampled, but urine could be collected at any time of the day in Groups 1 and 3 (“spot samples”). Measurements of biomarkers in urine sampled at any time of the day have slightly poorer precision than morning urine and 24-h urine for indicating 24-h urine output, but the accuracy for groups is acceptable or even good [25, 46]. The ability to sense thirst is impaired in the elderly [47–49], which might promote dehydration. Thirst was only assessed in 208 participants, but the those with concentrated urine (urine osmolality > 760 osmol/kg) showed not higher but lower thirst ratings compared to others. Hence, impaired thirst sensation might promote poor hydration in those who are middle-aged or older, although increased thirst is clearly a sign of dehydration in 20-year-olds [50].

Only two biomarkers were reported, although some of the cross-sectional studies also include measurements of urine-specific gravity and urine color. No composite dehydration tool was used, such as the Body Weight, Urine Color, and Thirst Level (WUT) [50]) or the Fluid Retention Index (FRI) [17, 19, 51]. Summarizing the result of multiple variables that all indicate dehydration could have provided a more robust test variable, but this was thought to be difficult to apply in a clinical setting.

## Conclusions

Measuring osmolality or creatinine in the morning urine of volunteers aged 23–62 years indicated whether the urinary excretion had been low on the previous day (< 1 L) with a sensitivity and specificity of approximately 80%. Application of the cutoffs to middle-aged hospital workers showed a high incidence of low urine output (approximately 50%) while the fraction decreased sharply with age in hospital patients. This suggests that the validity of the urine test should be re-evaluated in the elderly.

### Electronic supplementary material

Below is the link to the electronic supplementary material.

Supplementary Material 1: Original data in the diet study. Lines 1–82 show the measurements performed during the first 4 days of the study, when water intake was habitual, and Lines 84–162 show the second period (Days 8–11) when the ingestion of water was increased

Supplementary Material 2: Original data in the cross-sectional studies

## Data Availability

The datasets supporting the conclusions of this article are available as Supplementary File 1.xls (diet study) and Supplementary File 2.xls (cross-sectional studies).

## Abbreviations

ROC: receiver operating characteristic
mosmol/kg: milliosmoles per kilo
SD: standard deviation
WUT: body weight, urine color, and thirst level
FRI: fluid retention index
